# Atypical visual processing in posttraumatic stress disorder^☆^

**DOI:** 10.1016/j.nicl.2013.08.009

**Published:** 2013-08-29

**Authors:** Christoph Mueller-Pfeiffer, Matthis Schick, Thomas Schulte-Vels, Ruth O'Gorman, Lars Michels, Chantal Martin-Soelch, James R. Blair, Michael Rufer, Ulrich Schnyder, Thomas Zeffiro, Gregor Hasler

**Affiliations:** a Department of Psychiatry and Psychotherapy, University Hospital, Zurich, Switzerland; b Center of Education and Research (COEUR), Psychiatric Services of the County of St. Gallen-North, Wil, Switzerland; c Department of Psychiatry, Massachusetts General Hospital and Harvard Medical School, Boston, MA, USA; d Center for MR-Research, University Children's Hospital, Zurich, Switzerland; e Institute of Neuroradiology, University Hospital, Zurich, Switzerland; f Department of Clinical Psychology, University of Fribourg, Switzerland; g Mood and Anxiety Disorders Program, National Institute of Mental Health, National Institutes of Health, Bethesda, MD, USA; h Neural Systems Group, Massachusetts General Hospital, Boston, MA, USA; i Psychiatric University Hospital, University of Bern, Bern, Switzerland

**Keywords:** Visual system, Ventral stream, Dorsal stream, fMRI, International Affective Picture System, Sensory perception

## Abstract

**Background:**

Many patients with Posttraumatic Stress Disorder (PTSD) feel overwhelmed in situations with high levels of sensory input, as in crowded situations with complex sensory characteristics. These difficulties might be related to subtle sensory processing deficits similar to those that have been found for sounds in electrophysiological studies.

**Method:**

Visual processing was investigated with functional magnetic resonance imaging in trauma-exposed participants with (N = 18) and without PTSD (N = 21) employing a picture-viewing task.

**Results:**

Activity observed in response to visual scenes was lower in PTSD participants 1) in the ventral stream of the visual system, including striate and extrastriate, inferior temporal, and entorhinal cortices, and 2) in dorsal and ventral attention systems (*P* < 0.05, FWE-corrected). These effects could not be explained by the emotional salience of the pictures.

**Conclusions:**

Visual processing was substantially altered in PTSD in the ventral visual stream, a component of the visual system thought to be responsible for object property processing. Together with previous reports of subtle auditory deficits in PTSD, these findings provide strong support for potentially important sensory processing deficits, whose origins may be related to dysfunctional attention processes.

## Highlights

• Investigation of visual processing in PTSD using a picture-viewing task during fMRI
• Alteration was found in the visual system responsible for object feature processing.
• Lower visual responsiveness in PTSD was not accounted for by stimulus valence.
• Altered visual processing in PTSD may be related to attentional dysfunction.

## 1 Introduction

Posttraumatic Stress Disorder (PTSD) is a serious condition that can develop in the aftermath of a traumatic event. The disorder has a substantial impact on quality of life and functioning (Zatzick et al., 1997). Despite therapeutic advances over the past two decades, PTSD remains a rather treatment refractory condition (Bradley et al., 2005). Current classification schemas, including DSM-5 (American Psychiatric Association, 2013) and ICD-10 (World Health Organization, 2010), define PTSD based on symptoms of persistent re-experiencing of traumatic memories, avoidance of stimuli reminiscent of the traumatic event, negative cognition and mood, and increased arousal. In addition to these core features, PTSD patients often present with a range of other symptoms such as dissociation, included now in DSM-5 as a PTSD subtype, and medically unexplained symptoms including pain, gastrointestinal complaints, chronic fatigue, and visual problems (Engel et al., 2000; Foa et al., 2006; McFarlane, 2010; Pacella et al., 2013; Trachtman, 2010; Wolf et al., 2012).

Many PTSD patients feel overwhelmed or insecure in situations with high levels of complex sensory input, including large crowds, heavy traffic, large cities, public transportation, or crowded shopping malls. Electrophysiological methods have identified sensory processing disturbances at early, relatively automatic processing stages (Ge et al., 2011; Holstein et al., 2010; Hunter et al., 2011) that are thought to underlie hyperarousal symptoms in PTSD (Clark et al., 2009; Newport and Nemeroff, 2000). Further evidence for visual system dysfunction in PTSD patients are reports of feeling flooded and overwhelmed by multiple, simultaneous sensory stimuli, and experiencing lights or noises as unusually intense (Stewart and White, 2008). These puzzling symptoms are not fully subsumed under the hyperarousal cluster in DSM-V (American Psychiatric Association, 2013), and PTSD patients frequently struggle to articulate and understand these phenomena.

Both electrophysiological and magnetoencephalographic studies have provided preliminary evidence for atypical visual processing following traumatic experiences, evidenced by reduced occipital responses to neutral or angry faces (Felmingham et al., 2003), and positive or negative scenes (Adenauer et al., 2010, 2011; Catani et al., 2009) in PTSD compared to trauma-exposed and non-trauma-exposed healthy individuals. Structural imaging studies have demonstrated reduced regional gray matter volume in visual cortex in both individuals with PTSD (Chao et al., 2012; Zhang et al., 2011) and adult survivors of child sexual abuse (Tomoda et al., 2009, 2012), suggesting the possibility of lasting macrostructural alterations in regions specialized for visual processing. However, functional imaging studies in PTSD employing visual stimuli have primarily focused on the contrast between processing of pictures with either emotional or neutral valence (Bremner et al., 1999; Brunetti et al., 2010; Chao et al., 2012; Fani et al., 2012; Hendler et al., 2003; Hou et al., 2007; Phan et al., 2006; Shin et al., 1997, 2005; Williams et al., 2006). Concerning visual cortex, these studies have yielded inconsistent results, showing lower (Fani et al., 2012), higher (Bremner et al., 1999; Williams et al., 2006) or comparable (Brunetti et al., 2010; Chao et al., 2012; Hendler et al., 2003; Hou et al., 2007; Phan et al., 2006; Shin et al., 1997, 2005) activity in PTSD in response to threat-related and trauma-related visual stimuli. Because these studies (with one exception discussed below) did not test general visual processing by contrasting picture with non-picture conditions, they may have failed to detect atypical, more general visual processing abnormalities in PTSD. In this fMRI study we asked PTSD patients and trauma-exposed healthy controls to view pictures with varying emotional contents and found substantial reductions in task related activity in the ventral visual processing stream, perhaps related to atypical modulation by both dorsal and ventral attention systems. Surprisingly, these reductions were unrelated to the pictures' emotional content.

## 2 Method

### 2.1 Participants

Participants were right-handed (Oldfield, 1971), trauma-exposed (meeting DSM-IV criteria A1) individuals with (N = 18) and without (N = 21) a current DSM-IV PTSD diagnosis as assessed using the Clinician-Administered PTSD Scale (CAPS) (Blake et al., 1995). A CAPS score of greater than 50 was required for PTSD participants and less than 34 for trauma-exposed controls. Trauma history was assessed using the CAPS, the trauma checklist from the Posttraumatic Diagnostic Scale (PDS) (Foa et al., 1997), and the Childhood Trauma Questionnaire (CTQ) (Bernstein et al., 2003). No PTSD participant had a current comorbid dissociative disorder assessed using the Structured Clinical Interview for DSM-IV Dissociative Disorders-Revised (SCID-D-R) (Steinberg, 1994). Current Axis I disorders, assessed using the Structured Clinical Interview for DSM-IV Axis I Disorders (SCID-I), are presented in Table 1. All participants were free of neurological or other major medical conditions. Two PTSD participants and two trauma-exposed controls had a history of mild traumatic brain injury according to standard criteria (Kay et al., 1993). No participant had substance dependence except for two PTSD participants who had suffered from alcohol dependence two and 15 years ago. Seven PTSD participants and one trauma-exposed control were currently medicated with antidepressants, including selective serotonin or noradrenaline reuptake inhibitors. Six PTSD participants were taking medication as follows: a non-opioid analgesic (N = 1), an antiretroviral (N = 1), thyroid substitutes (N = 2), a calcium channel blocker (N = 1), and an anti-asthmatic (N = 1). One trauma-exposed control took an angiotensin II receptor antagonist.

Participants with PTSD were recruited from the psychiatric outpatient department of the University Hospital of Zurich and the Psychiatric Services of the County of St. Gallen-North, Switzerland, from individual local psychotherapists, and by advertisement. Trauma-exposed controls were recruited by advertisement.

Prior to scanning, participants completed the CTQ (Bernstein et al., 2003), Multidimensional Inventory of Dissociation (MID) (Dell, 2006), the trait portion of the State–Trait Anxiety Inventory (STAI) (Spielberger et al., 1970) and the Beck Depression Inventory (BDI) (Beck et al., 1961). Standard cognitive tests were administered using Hogrefe Test System 4 software (Hogrefe, 2006) and included the Viennese Matrices Test (Formann and Piswanger, 1979), an adapted version of the Raven Progressive Matrices (Raven, 1947), the Test of Word Power (Schmidt and Metzler, 1992), and the d2 Test of Attention (Brickenkamp and Zillmer, 1998). Immediately prior to scanning, participants completed the state portion of the STAI (Spielberger et al., 1970). All measures were German-adapted and validated versions. Socio-demographic and clinical data are presented in Table 1. The study protocol was approved by the Institutional Review Board of the County of Zurich, Switzerland. This study was carried out in accordance with the Declaration of Helsinki, and all participants provided written informed consent after full explanation of the procedures.

### 2.2 Task procedures

The participants engaged in a picture viewing task in which they were instructed to press a button when a picture containing a human being or human body part was shown. This response requirement was included only to direct the participant's attention to the presented pictures and to prevent behavioral avoidance such as eye closing. The task was not designed to investigate cognitive performance; consequently, response speed was not emphasized in the participants' instructions. Using images containing humans or human body parts as targets was motivated by practical reasons, because these targets could be unambiguously and easily categorized.

A total of 48 IAPS pictures spanning a range of emotional content (valence: mean = 4.8, SD = 2.1, range = 1.7–8.3; arousal: mean = 4.8, SD = 1.8, range = 1.7–7.3) were presented. Each of three identical sessions consisted of two sequences comprising non-repeating IAPS pictures with comparable normative ratings for valence and arousal. Each sequence consisted of three 30 s blocks containing neutral, positive, or negative pictures, with each block separated by a 30 s fixation point. In each block, 8 different pictures were presented for 400 ms with each picture presented twice in rapid succession (inter-stimulus interval = 400 ms) to make the stimulus more “salient”, followed by a variable inter-trial-interval of 2300–2800 ms. Thus, even though we utilized homogeneous stimulus blocks, the subsequent statistical modeling was consistent with rapid event related designs.

At the end of each sequence, cognitive and emotional self-reports referencing the previous task were collected using a 5-point Likert scale (“not at all” to “very much so”) with one item each for hypervigilance (“I felt vigilant”), numbing (“I felt emotional numb”), re-experiencing (“I experienced a flashback”), depersonalization (“My body felt vague, indefinite, strange”), derealization (“I felt far away from what was happening around me”) and somatoform dissociation (“I was unusually weak or paralyzed in one or more of my muscles”). The hypervigilance and numbing items were constructed according to DSM-IV PTSD criteria; the remaining four items were selected and adapted from the State Scale of Dissociation (Krüger et al., 2002), a 56-item scale that measures distinct dimensions of state dissociation, according to the results of a validation study of the German adaptation of the scale (Mueller-Pfeiffer and Wittmann, 2013). In contrast to the SSD, which uses a 10-point scale, we used a 5-point scale in order to allow collection of responses in the scanner using a 5-digit response unit (Fiber Optic Button Response System, Psychology Software Tools, Inc., Pittsburgh, PA).

After the picture viewing fMRI session, the IAPS pictures were again presented to the participants outside the scanner and they were asked to rate the emotional valence and arousal of each picture using the Self-Assessment Manikin, a 9-point, non-verbal pictorial assessment technique for measuring affective reactions to stimuli (Bradley and Lang, 1994). The task was implemented using E-Prime Professional 2.0 (Psychology Software Tools, 2010) and presented using video goggles.

### 2.3 Acquisition of MRI data

The participants were studied using a General Electric Signa HD.xt 3.0 T MR scanner with 8-channel receive-only head coil, located at the Center for MR-Research at the University Children's Hospital Zurich in Switzerland. Task-related activity estimates were obtained using an echo-planar imaging (EPI) sequence with repetition time = 3000 ms, echo time = 23 ms, 64 × 64 matrix, flip angle = 82°, and field of view = 24 cm. Whole brain coverage was obtained with 36 axial slices (thickness = 3.5 mm; in-plane resolution = 3.75 × 3.75 mm). A high-resolution anatomical scan covering the whole brain (three-dimensional spoiled gradient-echo (SPGR) with repetition time = 10.9 ms, echo time = 4.6 ms; field of view = 24 cm; flip angle = 13°; 156 axial slices; thickness = 1.2 mm; 352 × 224 matrix) was collected for voxel-based morphometry analysis.

### 2.4 Data analysis

#### 2.4.1 Analysis of behavioral data

For the analysis of clinical measures we used Fisher's Exact Test to compare proportions of nominal variables, and *t*-tests to compare continuous variables between groups. Performance indicators included response time and accuracy calculated from the proportion of errors. Missing responses were counted as errors. Response time and accuracy were examined separately with repeated measures generalized linear regression, using Gaussian and binomial models respectively, with group (PTSD, trauma-exposed controls), and IAPS valence and arousal scores as predictors. The critical threshold was *P* = 0.05 (two-sided). Statistical analyses of behavioral data were performed using R V.2.14.1 (R Development Core Team, 2011).

#### 2.4.2 Analysis of fMRI data

EPI preprocessing included: (1) realignment for head motion correction, (2) spatial normalization into the Montreal Neurological Institute (MNI) anatomical space, and (3) spatial smoothing of 8 mm full width at half maximum. Outliers in EPI time series were identified using the Artifact Detection Tools (www.nitrc.org/projects/artifact_detect/). For statistical analysis, we used a summary statistic approach comprising event-related models for each participant, followed by group mixed effects models using SPM8 (Wellcome Trust Centre for Neuroimaging, 2009).

At the first level, for each participant and session, IAPS picture onset times were used to construct an effect of interest regressor with the intervening fixation periods serving as an implicit baseline. Stimulus onset times of IAPS pictures, independent of their emotional content, were used to specify this regressor. In order to model the influence of picture emotional content, trial-specific IAPS picture valence and arousal scores were included as orthogonalized parametric modulators of task-related neural activity. We also explored an alternative approach to the modeling of emotional effects using separate regressors for neutral, negative and positive picture conditions, allowing estimation of between group effects specifically in the neutral picture condition. These results are reported in Supplemental Fig. 4.

The regressors for IAPS picture onset times and their parametric modulators, together with other regressors modeling head movement, the mean signal for the session, outliers, and a discrete cosine transform basis set modeling the low-frequency, presumably artifactual, signal modulations (cut-off 1/128 Hz), jointly comprised the full model for each participant. Parameter estimates for each regressor were calculated from the fit of the model to the data using restricted maximum likelihood algorithms.

At the second level, mixed-effects analyses included the three contrast images resulting from the first level model estimates, representing activity associated with: (1) the picture viewing condition, and (2) IAPS valence and arousal parametric modulators. Processing of IAPS pictures, and the parametric modulation by picture valence and arousal scores were examined with separate between-group two-sample *t*-tests (one-sided). Effect of task speed was examined by inclusion of mean reaction time as a covariate in a separate second level model. Habituation in picture-related activity and modulation of picture-related activity by picture valence and arousal scores were examined using separate omnibus *F*- and planned *t*-contrasts in conjunction with a two (group: PTSD participants, trauma-exposed controls) by three (session: 1, 2, 3) ANOVA. Cohen's effect sizes (*d*) were calculated from the results of the planned *t*-contrasts. The relationship of task-related activity to sociodemographic status and psychopathology in PTSD subjects was determined by including sociodemographic and clinical measures as covariates in separate second level models. For convenience of interpretation, Pearson's *r*-values were derived from corresponding *t*-contrasts for these covariates. The critical threshold for within- and between-group voxel-wise estimates of task-related activity peaks was *P* = 0.05, whole-brain, family-wise error (FWE)-corrected, providing strong protection from Type I error.

## 3 Results

### 3.1 Behavioral results

PTSD participants responded on average 203 ms (95% CI [29, 377]) more slowly to pictures than trauma-exposed controls (*P* = 0.022). There were no significant group differences regarding accuracy (*P* = 0.064). PTSD participants rated pictures on average 6.7% (95% CI [2.2, 11.3]) less pleasant (*P* = 0.007) and 11.4% (95% CI [2.4, 20.3]) more arousing (*P* = 0.012) than trauma-exposed controls. Higher arousal ratings of IAPS pictures were associated with lower accuracy in both groups (*P* < 0.001). There were no significant between-group differences in picture valence and arousal on speed or accuracy. While in the MRI system PTSD participants reported significantly higher hypervigilance, numbing, re-experiencing, depersonalization, derealization, and somatoform dissociation than trauma-exposed controls (*P*s < 0.001). Behavioral results are presented in Supplemental Table 1 and Supplemental Fig. 1.

### 3.2 FMRI results

#### 3.2.1 Viewing IAPS pictures

**Fig. 1:**
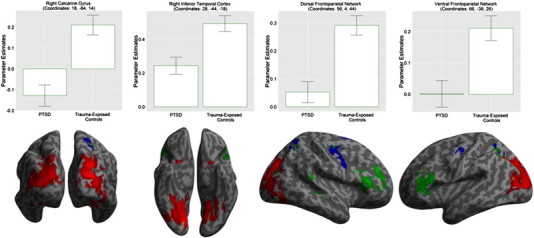
Group differences in response to viewing IAPS pictures in trauma-exposed controls (N = 21) versus PTSD participants (N = 18). There was lower activity in response to pictures compared to baseline in PTSD participants compared to trauma-exposed controls in the ventral visual stream (red); and the dorsal frontoparietal (blue) and ventral frontoparietal networks (green) of the attention system. The bars represent parameter estimates relative to the mean across conditions (baseline); the vertical bars show 90% confidence intervals. Effects exceeded a critical threshold of *P* = 0.05, FWE-corrected; clusters are presented here at *P* < 0.001, uncorrected. PTSD: Posttraumatic Stress Disorder; IAPS: International Affective Picture System.

Brain regions that showed significantly lower (*P* < 0.05, FWE-corrected) activity in response to IAPS pictures in PTSD participants compared to trauma-exposed controls are listed in Table 2. The listed brain regions generally belong to one of three brain *systems* (Fig. 1): (1) *visual* regions, specifically ventral stream regions including striate, extrastriate, inferior temporal, and entorhinal cortices, (2) *dorsal attention* regions including supplementary motor area, precentral gyrus, and superior parietal lobule and (3) *ventral attention* regions including middle and inferior frontal gyri, and inferior parietal lobule. Activity related to picture processing for each group is presented in Table 3 and Supplemental Fig. 2. There were no regions with significantly greater activity in response to pictures in the PTSD group. Participants' valence and arousal ratings had no effect on picture-related activity, either in participants with PTSD or in trauma-exposed controls. Lower task speed did not account for the decreased visual activity in PTSD participants. Using a cluster level FWE-corrected critical threshold, participants in both groups showed higher activity in response to pictures in the first compared to the second session in right inferior parietal lobule (coordinates: 46, − 44, 36; *t* = 5.03), right middle frontal gyrus (coordinates: 38, 26, 44; *t* = 4.88), right superior temporal gyrus (coordinates: 56, − 34, − 14; *t* = 4.36), left precentral gyrus (coordinates: − 46, 10, 44; *t* = 4.31), and left inferior temporal gyrus (coordinates: − 50, − 54, − 22; *t* = 4.29). There was no session by group interaction, providing no evidence against similar habituation effects in both groups. There was no habituation in participants' valence and arousal rating effects on picture-related activity, either in participants with PTSD or in trauma-exposed controls.

#### 3.2.2 Additional analyses

Using whole-brain FWE-correction, we did not find any association between visual cortical activity and either sociodemographic or psychometric measures in PTSD participants. In exploratory analyses using an uncorrected critical threshold (*P* < 0.001), we found an association between higher CAPS total scores and lower activity in striate (right lingual gyrus; coordinates: 20, − 88, − 2; *r* = -0.77) and extrastriate cortices (left middle occipital gyrus; coordinates: − 16, − 92 − 4; *r* = -0.70).

In order to control for confounding effects of medication, we repeated the analysis of picture viewing in participants free of psychotropic medication (11 PTSD participants, 20 trauma-exposed controls). In this subsample we again found lower picture-related activity in PTSD participants in striate and extrastriate cortices (*P* < 0.05, FWE-corrected; Supplemental Fig. 3), suggesting that psychotropic medication did not account for the observed lower visual cortex activity in PTSD participants.

## 4 Discussion

In this study, we observed lower activity in regions of the ventral visual stream responsible for object feature processing in PTSD participants compared to non-PTSD trauma-exposed controls while viewing scenes drawn from the IAPS picture set. Lower visual responsiveness in PTSD participants was not accounted for by the emotional content of the pictures. Lower activity was also seen in both dorsal and ventral attention systems, suggesting that the atypical visual processing may be related to attentional dysfunction. These novel neuroimaging findings extend previous observations of deficits in auditory processing in PTSD (Clark et al., 2009) and suggest strategies for more effective treatments.

Our results are consonant with prior event-related potential (Felmingham et al., 2003) and magnetoencephalographic studies (Adenauer et al., 2010, 2011; Catani et al., 2009) that found reduced occipital responses in PTSD participants to neutral and emotional stimuli during picture viewing tasks. Sensory processing deficits in PTSD have been seen in electrophysiological and imaging studies showing enhanced auditory sensitivity (Bryant et al., 2005), and, at an early temporal stage in the processing stream, excessive auditory novelty detection (Morgan Iii and Grillon, 1999), and difficulties in filtering and discriminating auditory stimuli (McFarlane et al., 1993; Skinner et al., 1999). In addition, reduced gray matter volume in visual cortex in trauma victims has been found in structural imaging studies (Chao et al., 2012; Tomoda et al., 2009; Zhang et al., 2011). However, using voxel-based morphometry in our sample we did not find any volumetric decreases in occipital cortex in PTSD participants (data not shown), suggesting that the visual processing differences we observed did not result from partial-volume effects. Taken together, there is considerable evidence for atypical sensory processing in PTSD.

A previous fMRI study (Phan et al., 2006) failed to detect visual cortex activity differences in response to neutral IAPS compared to blank pictures in war veterans with PTSD, war veterans without PTSD and non-combat controls. These discrepant findings may be caused by methodological differences between the studies, for example, the use of longer stimulus presentation times of 5 s compared to the shorter 400 ms presentations in our study.

Visual analysis in humans and mammals is believed to consist of multiple stages that are organized in parallel and hierarchical processing streams. The early stages involve transforming the visual stimulus into neural activity patterns that are transmitted via the retina and the lateral geniculate nucleus to the striate visual cortex (V1), an area responsible for the analysis of simple visual features (e.g., lines, colors). In the later stages, visual information is distributed from V1 to neighboring occipital, parietal and temporal regions, specialized for processing additional features of increasing complexity. These projections can be divided in a dorsal stream related to object location and movement, and a ventral stream related to object recognition (Ungerleider and Bell, 2011). In agreement with reported structural PTSD abnormalities (Chao et al., 2012; Tomoda et al., 2009; Zhang et al., 2011), we found the most prominent reduction in neural activity in PTSD participants in the ventral stream of the visual system including striate and extrastriate visual cortices, ventral occipital cortex, inferior temporal cortex, and entorhinal cortex. This network has been found to mediate the identification and recognition of complex visual features such as faces, scenes, and body parts (Spiridon et al., 2006), their spatial representation (Killian et al., 2012), and visual memory (Brewer et al., 1998).

Atypical activity in the visual system in PTSD might result from local dysfunction. Transmarginal inhibition, a “shutting-down” response of the nervous system to overwhelming stimuli (Pavlov, 1927), might explain lowered processing by a hypersensitized visual system in PTSD. Alternatively, or in addition, there might be atypical influences on visual processing from attention mechanisms that are required to select the most relevant objects from among the many features competing for limited visual processing resources (Desimone and Duncan, 1995). Atypical attention allocation to visual stimuli in PTSD is suggested by our observation of lower activity in PTSD participants in the dorsal and ventral frontoparietal networks, which are believed to play an important role in voluntarily focusing attention to current behavioral goals and involuntarily orienting to novel stimuli (Corbetta et al., 2008). Because we did not manipulate attention in this study, this possible mechanism will require further investigation, and is in apparent conflict with our findings from the d2 Test of Attention (Table 1), where PTSD participants performed similarly to trauma-exposed controls. However, it might be that atypical attention allocation manifests in the presence of complex visual scenes, such as IAPS pictures, but not in less complex visual stimuli such as letters and dashes, used in the d2 Test of Attention. Moreover, it cannot be excluded that the MRI environment, including loud noise and uncomfortable head fixation might have an adverse impact on attention regulation in participants with partially compensated attention skills.

The lack of emotional modulatory effects on cortical visual activity we observed in PTSD participants is in agreement with some (Phan et al., 2006; Shin et al., 2005) but not all (Brunetti et al., 2010; Chao et al., 2012; Williams et al., 2006) previous PTSD studies employing trauma-unrelated negative scenes or facial affect pictures in visual tasks. In contrast to our study, most (Brunetti et al., 2010; Phan et al., 2006; Shin et al., 2005; Williams et al., 2006) but not all (Chao et al., 2012) studies found altered activity in medial or lateral prefrontal cortex, medial or lateral prefrontal cortex, anterior cingulate cortex or amygdala in response to negative versus neutral stimuli in PTSD. The lack of an emotional modulatory effect in PTSD participants in the present study might be explained by generally less efficient visual processing, which could be associated with a need for stronger emotional stimuli, such as trauma-related cues, to elicit typical cortical activity levels (Rauch et al., 1996). As an alternative, it is possible that brief, almost subliminal, stimulus presentation could make engagement of cognitive avoidance strategies less likely (Hendler et al., 2003). Similarly, we observed no emotional modulatory effect in visual cortex activity in trauma-exposed controls, a finding that agrees with most (Kosslyn et al., 1996; Phan et al., 2006; Williams et al., 2006) but not all (Brunetti et al., 2010; Chao et al., 2012; Shin et al., 2005) previous work. Again, and in contrast to our results, most of these studies reported an effect of emotion in areas other than visual cortex. Differences in sample characteristics (all of the studies cited above involved combat veterans, firefighters, and survivors of non-sexual assault or accidents) or task characteristics, including stimulus presentation duration and repetition rate (Rotshtein et al., 2001) might account for these discrepant findings in both PTSD participant and trauma-exposed controls.

We observed greater picture related activity in the first compared to the second session in right inferior parietal lobule, right middle frontal gyrus, and left precentral gyrus. Given that these regions are believed to be involved in attention (Corbetta et al., 2008), invoking an explanation referencing habituation effects during task repetition seems reasonable, as the same set of pictures was used in all sessions. Since precentral gyrus is also part of the motor system, activity decrease across sessions might also result from behavioral habituation or motor fatigue (Rankin et al., 2009). As expected, activity decrease across sessions was observed in the ventral visual stream (Miller et al., 1991).

In agreement with our behavioral results, many previous studies employing visual selective or sustained attention tasks, reported impaired performance including higher reaction times in PTSD (e.g., Jenkins et al., 2000) (for a review, see Aupperle et al., 2012). However, our intention was not to measure cognitive performance, as speed was not emphasized and the task had low cognitive demands. Hence, we cannot exclude possible effects from other psychological factors, including possible greater efforts made by PTSD participants to avoid false responses, accounting for slower response times.

A limitation of this study is that the use of antidepressants by some participants might have influenced the results. However, repeating the between-group analyses after exclusion of participants (N = 8) taking psychotropic medication revealed similar findings. We did not include an additional trauma-unexposed comparison group, which does not allow us to make inferences regarding the specific visual processing consequences of traumatic experiences not leading to subsequent PTSD. We also cannot exclude the possibility that our results are influenced by context effects. For instance, PTSD participants might have been threatened by the fMRI testing environment, leading to heightened anxiety and associated difficulties maintaining attention to the pictures, and thereby influencing reaction times and activity in visual areas. As inclusion of reaction time in the model did not account for the between group differences, this explanation seems unlikely. Another potential explanation for the lower visual activity we observed in PTSD is the experience of dissociation during the experiment. However, we found neither a significant correlation between dissociation levels and neural activity, nor higher activity in medial prefrontal areas in PTSD participants, as has been postulated in the corticolimbic inhibition model for dissociative PTSD (Lanius et al., 2010). It is unlikely for two reasons that lower activity in the visual system represents simple avoidance of attending to IAPS pictures. First, the stimuli were presented with closed video glasses, which do not allow overt orienting of attention outside the context of the experiment. Second, we did not find higher activity in the frontal eye fields in PTSD participants, of the type that would be associated with more eye movements in participants not focusing on the center of the pictures but generating more avoidance saccades. Other behavioral avoidance strategies such as eye closure would have seriously impaired behavioral performance. Since a majority of our participants were females, any generalization of our results to males with PTSD must be drawn with caution. Finally, our results do not resolve the issue of whether the abnormalities we discovered reflect a risk factor for, or a consequence of, PTSD. In order to further investigate this question, prospective or twin pair studies will be required.

Experimental strengths of this study include the persistence of the main results after controlling for potential emotion effects, and after conservative whole-brain correction for multiple comparisons, making Type I errors unlikely. A further strength is the comparable trauma history between PTSD participants and the trauma-exposed comparison group.

In summary, our results document atypical visual processing in PTSD of a very basic sort, involving picture processing. The findings extend prior evidence for atypical auditory processing in PTSD, suggesting that the pathophysiological locus may be independent of specific sensory modality. The observed subtle deficits in sensory processing might explain difficulties that PTSD patients have with complex sensory environments, even in the absence of emotional interference. Further research will explore whether local dysfunction in the visual system and/or the cortical network responsible for directing attention toward relevant visual cues, is primarily responsible for diminished visual processing in PTSD. Moreover, the development of specific tests for measuring sensory deficits in PTSD may provide important new data allowing development of neurobiological models better explaining the multi-domain nature of non-specific PTSD symptoms that are currently major issues of controversy and interest (McFarlane, 2010).

In a more speculative vein, this study suggests possible avenues for developing new therapeutic approaches for PTSD. For instance, mindfulness-based therapy, which incorporates self-regulation of attention (Bishop et al., 2004), is effective in the treatment of anxiety (Hofmann et al., 2010) and is generally considered a useful second-line approach in the treatment of complex PTSD (Cloitre et al., 2011). It is possible that part of the mechanism by which mindfulness therapy is helpful in PTSD is related to its modulation of selective attention related to orienting to salient visual stimuli. Given the potential adverse impact of attention disturbances on sensory processing, further research should investigate whether intensive attention-based interventions might enhance the outcome of current first-line treatment for PTSD such as cognitive and behavioral trauma-exposure therapies.

## Figures and Tables

**Table 1:** Demographic and clinical characteristics of PTSD participants and trauma-exposed controls.

	Group				
	PTSD (N = 18)		Trauma-exposed controls (N = 21)		Analysis
Measure	N	%	N	%	*P*
Female	17	94.4	18	85.7	0.609
Current Axis I comorbidity					
Depressive disorder	6	33.3	0	0	
Dysthymia	2	11.1	0	0	
Panic disorder	2	11.1	0	0	
Agoraphobia	1	5.7	0	0	
Social phobia	3	16.7	0	0	
Specific phobia	2	11.1	0	0	
Obsessive–compulsive disorder	3	16.7	0	0	
Generalized anxiety disorder	1	5.7	0	0	
Body dysmorphic disorder	1	5.7	0	0	
Bulimia nervosa	1	5.7	1	4.8	
Type index trauma					0.710
Accident	5	23.8	3	14.3	
Medical event	1	4.8	1	4.8	
Rescue worker	1	4.8	1	4.8	
Natural disaster	0	0.0	2	9.5	
Single physical assault	4	19.0	2	9.5	
Single sexual assault	2	9.5	2	9.5	
Childhood physical/sexual abuse	3	14.3	4	19.0	
Combat trauma	1	4.8	4	19.0	
Intimate partner violence	1	4.8	2	9.5	
Medication					
Antidepressant	7	38.9	1	4.8	0.015
For physical medical conditions	6	33.3	1	4.8	0.003

	Group				

PTSD (N = 18)		Trauma-exposed controls (N = 21)		Analysis

Measure	Mean	SD	Mean	SD	*P*

Age (years)	37.3	12.3	36.7	11.1	0.869
Education (years)	14.1	3.7	15.2	3.3	0.342
EHI: Right handedness	13.9	5.0	13.7	1.0	0.844
EHI: Left handedness	2.2	3.3	1.7	2.9	0.620
Cognitive performance					
d2: Total number of items processed (processing speed)	460.6	75.0	510.2	93.6	0.074
d2: Total number of errors (accuracy)	7.7	5.6	11.0	11.1	0.251
WMT: Total number of correct responses (non-verbal intelligence)	12.8	4.2	13.3	5.2	0.762
WST: Number of recognized words (verbal intelligence)	31.4	5.3	33.2	2.8	0.227
CAPS: Total	72.8	12.9	5.5	6.6	< 0.001
CAPS: Re-experiencing	22.8	5.9	2.4	3.4	< 0.001
CAPS: Avoidance	27.0	9.3	1.1	2.2	< 0.001
CAPS: Hyperarousal	23.0	4.3	2.1	3.5	< 0.001
PDS: Number of self-reported single trauma	1.8	1.2	1.6	0.9	0.447
PDS: Number of self-reported prolonged and repeated trauma	0.8	1.3	0.5	0.8	0.386
Duration since index trauma	5.3	5.8	8.3	9.8	0.268
CTQ: Emotional abuse	15.1	7.2	10.1	5.4	0.024
CTQ: Physical abuse	9.0	4.6	7.5	3.9	0.278
CTQ: Sexual abuse	8.5	5.6	6.9	3.3	0.298
CTQ: Emotional neglect	15.9	7.2	11.2	5.8	0.033
CTQ: Physical neglect	10.7	5.5	7.2	2.6	0.024
MID: Total	18.5	12.9	1.8	2.3	< 0.001
STAI: State anxiety	51.6	10.3	29.0	5.6	< 0.001
STAI: Trait anxiety	55.9	10.0	32.7	10.0	< 0.001
BDI: Total	27.7	12.2	6.3	4.5	< 0.001

PTSD: Posttraumatic Stress Disorder; EHI: Edinburgh Handedness Inventory; d2: d2 Test of Attention; WMT: Viennese Matrices Test; WST: Test of Word Power; CAPS: Clinician-Administered PTSD Scale; PDS: Posttraumatic Diagnostic Scale; CTQ: Childhood Trauma Questionnaire; MID: Multidimensional Inventory of Dissociation; STAI: State–Trait Anxiety Inventory; BDI: Beck Depression Inventory.

**Table 2:** Brain regions showing higher activity in response to viewing IAPS pictures in trauma-exposed controls versus PTSD participants.

Region^a^	MNI coordinates			Analysis	
	x	y	z	*t*	Cohen's *d*
*Trauma-exposed controls > PTSD participants*					
L calcarine gyrus (striate visual cortex)	− 14	− 66	6	6.95	1.32
R calcarine gyrus (striate visual cortex)	18	− 64	14	8.02	1.53
R middle occipital gyrus (extrastriate visual cortex)	26	− 86	14	7.41	1.41
L middle occipital gyrus (extrastriate visual cortex)	− 22	− 92	12	6.90	1.32
R lingual gyrus (extrastriate visual cortex)	8	− 72	− 10	7.32	1.39
L lingual gyrus (extrastriate visual cortex)	− 16	− 50	− 6	6.55	1.25
R inferior temporal cortex	28	− 44	− 18	5.91	1.13
L entorhinal cortex	− 22	10	− 14	6.07	1.16
R entorhinal cortex	18	10	− 14	7.07	1.35
R supplementary motor area (BA6)	10	4	64	5.69	1.08
R precentral gyrus (BA6)	56	4	44	7.67	1.46
L postcentral gyrus (BA4)	− 42	− 16	40	5.34	1.02
R middle frontal gyrus	42	44	28	5.69	1.09
L inferior frontal gyrus (pars triangularis; BA45)	− 54	26	26	5.12	0.98
R superior parietal lobule (precuneus)	16	− 48	52	5.18	0.99
R inferior parietal lobule (supramarginal gyrus)	66	− 38	26	5.94	1.13
R hippocampus	12	− 38	8	6.12	1.17

a Peak voxels of regions with a whole-brain FWE-corrected *P*-value less than 0.05 and an extent threshold of *κ* = 10 voxels are reported. IAPS: International Affective Picture System; PTSD: Posttraumatic Stress Disorder.

**Table 3:** Brain regions showing higher activity in response to viewing IAPS pictures in PTSD participants compared to trauma-exposed controls.

Region^a^	MNI coordinates			Analysis	
	x	y	z	*t*	Cohen's *d*
*Pictures > baseline (mean across conditions) in PTSD participants*					
L fusiform gyrus^b^	− 40	− 42	− 22	12.68	2.42
R fusiform gyrus^b^	32	− 52	− 14	12.13	2.31
R supplementary motor area	14	26	66	6.00	1.14
L precentral gyrus (BA6)	− 42	− 4	62	7.56	1.44
R precentral gyrus	44	0	44	7.16	1.36
L postcentral gyrus (BA6)	− 24	− 34	78	5.64	1.08
R middle frontal gyrus	46	14	54	7.16	1.37
R inferior frontal gyrus (pars opercularis)	36	4	34	7.09	1.35
L superior parietal lobule	− 26	− 62	44	6.30	1.20
L inferior parietal lobule	− 30	− 54	46	6.96	1.33
R inferior parietal lobule	46	− 44	46	5.83	1.11
R inferior parietal lobule (angular gyrus)	32	− 58	50	6.85	1.31
L middle temporal gyrus	− 56	− 54	8	5.89	1.12

*Pictures > baseline (mean across conditions) in trauma-exposed controls*					
R middle occipital gyrus^b^	26	− 90	18	20.97	4.00
L middle occipital gyrus^b^	− 20	− 94	12	19.49	3.27
L supramarginal gyrus^c^	− 60	− 46	28	5.15	0.98
R supramarginal gyrus^c^	64	− 42	26	10.66	2.03
R middle frontal gyrus^d^	44	44	28	12.70	2.42
L middle frontal gyrus d	− 34	40	38	7.15	1.36
R hippocampus	22	2	− 32	5.06	0.96

a Peak voxels of regions with a whole-brain FWE-corrected *P*-value less than 0.05 and an extent threshold of *κ* = 10 voxels are reported.

b Cluster extends to striate and extrastriate visual cortices.

c Cluster extends to large portions of parietal lobe.

d Cluster extends to large portions of frontal lobe. IAPS: International Affective Picture System; PTSD: Posttraumatic Stress Disorder.
