# Supplementary material for: Atypical visual processing in posttraumatic stress disorder
Source: Neuroimage Clin. 2013 Aug 29;3:531–8. doi: 10.1016/j.nicl.2013.08.009 (PMC3871398; doi:10.1016/j.nicl.2013.08.009)
Supplement: Supplementary file 1 — Supplementary material. [file mmc1.docx]

### Supplemental Material

| **Supplemental Table 1** Task performance and rating of IAPS pictures in PTSD participants and trauma-exposed controls. | | | | | | | | |  |
| --- | --- | --- | --- | --- | --- | --- | --- | --- | --- |
|  | Group | | | | | Analysis | | |  |
|  | PTSD  (N=18) | | Trauma-Exposed  Controls  (N=21) | | |  | | |  |
| Measure | Mean | SD | | Mean | SD |  |  | *P* | |
| Task performance |  |  | |  |  |  |  |  | |
| Response time (ms) | 564.0 | 342.5 | | 322.9 | 206.8 |  |  | 0.022 | |
| Accuracy (%) | 84.9 | 21.8 | | 82.7 | 31.2 |  |  | 0.064 | |
| Rating of IAPS pictures |  |  | |  |  |  |  |  | |
| Valence | 4.7 | 0.6 | | 5.3 | 0.6 |  |  | 0.007 | |
| Arousal | 5.1 | 0.9 | | 4.0 | 1.5 |  |  | 0.012 | |
| IAPS: International Affective Picture System; PTSD: Posttraumatic stress disorder. | | | | | | | | | |

| 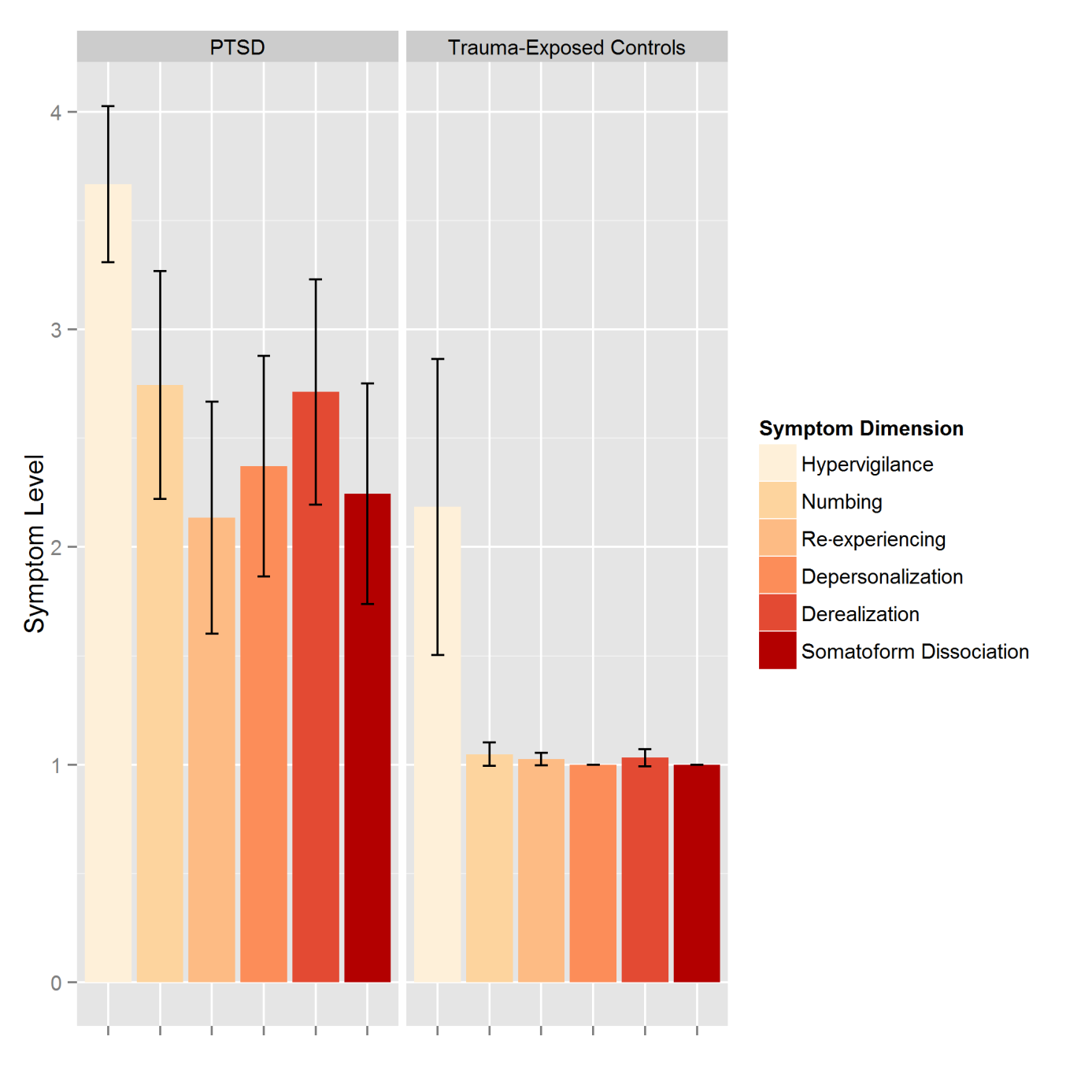 |
| --- |
| **Supplemental Fig. 1.** Cognitive and emotional self-reports during a picture-viewing task in PTSD participants and trauma-exposed controls. The bars represent mean symptom level scores; the vertical bars represent 95% confidence intervals. PTSD participants reported significantly higher hypervigilance, numbing, re-experiencing, depersonalization, derealisation, and somatoform dissociation than trauma-exposed controls (*P*s<0.001). PTSD: Posttraumatic stress disorder. |

| A | **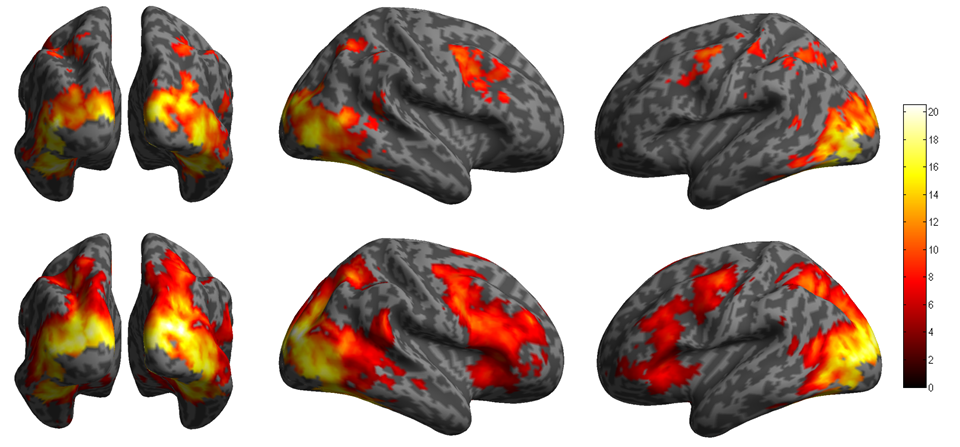** |
| --- | --- |
| B |  |
|  |  |
| **Supplemental Fig. 2.** Viewing IAPS pictures in PTSD participants and trauma-exposed controls was associated with widespread cortical activity increases. We observed increased activity in response to pictures compared to baseline in both: (A) PTSD participants and (B) trauma-exposed controls in bilateral striate cortex and extrastriate cortex, and dorsal and ventral frontal and parietal regions comprising attention processing networks. The color scale shows associated *t*-statistics. Effects in presented regions exceeded a critical threshold of *P*=0.05, FWE-corrected. IAPS: International Affective Picture System; PTSD: Posttraumatic stress disorder. | |

| 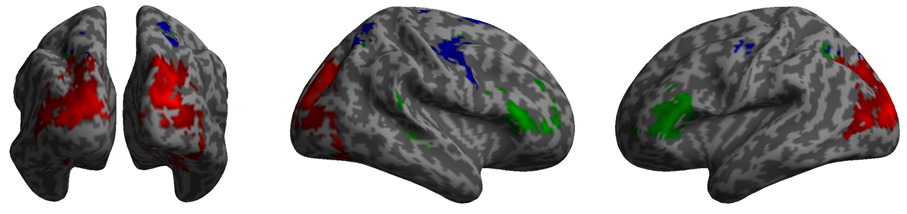 |
| --- |
| **Supplemental Fig. 3.** Lower activity in response to viewing IAPS pictures in PTSD participants (N=11) compared to trauma-exposed controls (N=20) free of psychotropic medication. Lower activity was observed in response to pictures compared to baseline in PTSD participants compared to trauma-exposed controls in bilateral striate and extrastriate cortex (red), dorsal frontoparietal network (blue), and ventral frontoparietal network (green). Effects exceeded a critical threshold of *P*=0.05, FWE-corrected; clusters are presented here at *P*<0.001, uncorrected. IAPS: International Affective Picture System; PTSD: Posttraumatic stress disorder. |

| A | **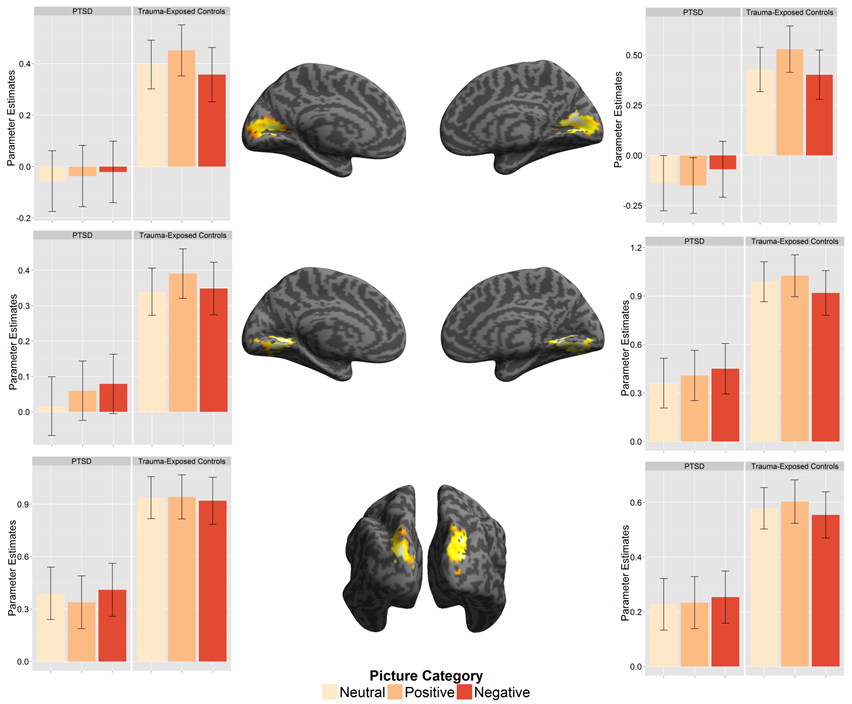** |
| --- | --- |
| B |  |
| C |  |
|  |  |
| **Supplemental Fig. 4.** Results of a block design analysis that included separate regressors for neural activity associated with neutral, positive, and negative IAPS pictures in the first level model. At the second level mixed-effects analyses were performed on contrast images from the first level representing the activity associated with the task conditions (neutral pictures, positive pictures, negative pictures), for each participant. We found a main effect of group in a two (group: PTSD participants, trauma-exposed controls) by three (picture category: neutral, positive, negative) ANOVA for activity in response to viewing IAPS pictures. There was lower activity in response to pictures across emotional content compared to baseline in PTSD participants compared to trauma-exposed controls in bilateral striate cortex (calcarine gyrus [A]) and bilateral extrastriate cortex (lingual gyrus [B]; superior occipital gyrus [C]). There was no significant group by picture category interaction effect. The bars represent parameter estimates relative to the mean across conditions (baseline); the error bars represent 90% confidence intervals. Effects in presented regions exceeded a critical threshold of *P*=0.05, FWE-corrected; clusters are presented here at *P*<0.001, uncorrected. IAPS: International Affective Picture System; PTSD: Posttraumatic stress disorder. | |
